# Supplementary material for: Biomathematical enzyme kinetics model of prebiotic autocatalytic RNA networks: degenerating parasite-specific hyperparasite catalysts confer parasite resistance and herald the birth of molecular immunity
Source: PLoS Comput Biol. 2025 Jan 3;21(1):e1012162. doi: 10.1371/journal.pcbi.1012162 (PMC11745417; doi:10.1371/journal.pcbi.1012162)
Supplement: S1 Definition of terms and notations — The model parameters (1st column), the values (2nd column) and a short textual explanation (3rd column) are listed for the main models and for the submodels. Reference to Methods or Models, and S1 Supporting Information is made where appropriate. (DOCX) [file pcbi.1012162.s001.docx]

**S1 Definition of terms and notations**

*Accuracy rate trade-off*: accuracy-rate trade-offs in enzymes relate to what degree increased accuracy leads to lower catalytic efficiency [1]. Accuracy is expressed as the ratio of *k_cat_/K_M_* values for the cognate substrate and alternative, non-cognate one(s) [1]. Accuracy can be achieved by two mechanisms, *ground-state discrimination* and *transition-state discrimination* [1].

*Degenerate:* degenerative process predicted to take place for parasitic templates, characterized by an accelerated rate of sequence and structural evolution with respect to the host molecule species [2-6].

*Diffusion rate limit*: the rate constant of encounters between an enzyme and its substrate. For ideal, perfect enzymes (few examples) diffusion on-rates of ∼10^9^ s^−1^ M^−1^ have been calculated, for average enzymes the typical *k_cat_/K_M_* values are ∼10^5^ s^−1^ M^−1^, indicating that the majority of encounters with substrates are futile [7].

*Ground-state discrimination*: it is driven by higher rate of association (*k_on_*), and/or lower dissociation rate (*k_off_*), of the cognate relative to the noncognate enzyme-substrate complexes [1]. Any improvement in selectivity derived from tighter binding of the cognate substrate reduces *k_cat_* by the same factor [1].

*Mathematical notation:* If not alternatively specified, n-vectors are always column-vectors. If a real valued column-vector $x\epsilon\mathbb{R}^{n}$is transposed to a row-vector we write$x^{T}$. Accordingly, also $nxm$ matrices $A\epsilon\mathbb{R}^{nxm}$ can be transposed by the same symbol $A^{T}$. The $i_{th}$entry of a vector is denoted by$x_{i}$. The entry of the $i_{th}$ row and the $j_{th}$ column of a matrix $A$ is denoted by $A_{ij}$.

*Michaelis-Menten equation* derived by Briggs and Haldane [8] using the *quasi-steady-state approximation*: the equation describes the dependence of enzyme-catalyzed reaction rates on the concentration of substrate by using two parameters, the catalytic constant, *k_cat_* and the Michaelis-Menten constant, *K_M_*. The *k_cat_* determines the maximum rate of the reaction at saturating substrate concentrations, *V_max_*=*k_cat_E_T_*, where *E_T_* is total enzyme concentration, and the *K_M_* is the substrate concentration at which the reaction rate is half of *V_max_* [8].

The *Michaelis– Menten equation* is schematically described as follows

$$E+S \begin{matrix} k_{on} \\ \rightleftharpoons\\ k_{off} \end{matrix}E-SE\_S \begin{matrix} k_{cat} \\ ⇀ \\ \end{matrix}E+P,$$

where *k_on_* and *k_off_* are the rates at which the enzyme *E* binds and unbinds the substrate *S*, respectively (6).

*Model parameters*

*Model 1:*

| ***Parameter*** | ***Values*** | ***Explanation*** |
| --- | --- | --- |
| $d_{R_{i}}$ | 0.005 | *decay rate (per unit of time) of ribozyme* $R_{i}, i=1,2$ |
| $K$ | $20.0$ | *maximum concentration of sum of ribozymes* $R_{1}+R_{2}$ |
| $\Pi$ | $\left( 1-\frac{R_{1}+R_{2}}{K} \right)$ | *habitat restriction term* |
| $k_{on}=\left[ \begin{matrix} k_{on}^{1,1} & k_{on}^{1,2} \\ k_{on}^{2,1} & k_{on}^{2,2} \end{matrix} \right]$ | $\left[ \begin{matrix} 0.2 & 0.19 \\ {10}^{-5} & {10}^{-5} \end{matrix} \right]$ | *parameter matrix for Model 1 (Methods or Models)* |
| $k_{off}=\left[ \begin{matrix} k_{off}^{1,1} & k_{off}^{1,2} \\ k_{off}^{2,1} & k_{off}^{2,2} \end{matrix} \right]$ | $\left[ \begin{matrix} 0.1 & 0.1 \\ 0.1 & 0.1 \end{matrix} \right]$ | *parameter matrix for Model 1 (Methods or Models)* |
| $k_{cat}=\left[ \begin{matrix} k_{cat}^{1,1} & k_{cat}^{1,2} \\ k_{cat}^{2,1} & k_{cat}^{2,2} \end{matrix} \right]$ | $\left[ \begin{matrix} 0.1 & 0.08 \\ 0.0 & 0.0 \end{matrix} \right]$ | *parameter matrix for Model 1 (Methods or Models)* |

*Model 2: (Model 2.1, Model 2.2)*

| ***Parameter*** | ***Values*** | ***Explanation*** |
| --- | --- | --- |
| $d_{R_{i}}$ | 0.005 | *decay rate (per unit of time) of ribozyme* $R_{i}, i=1,2,3$ |
| $q_{R_{3}}$ | 2.0 | *factor quantizing replication efficiency of parasites* $R_{3}$*relative to host ribozymes* $R_{1},R_{2}$ |
| $K$ | $20.0$ | *maximum concentration of weighted sum of ribozymes* $R_{1}+R_{2}+\frac{R_{3}}{q_{R_{3}}}$ |
| $\Pi$ | $\left( 1-\frac{R_{1}+R_{2}+\frac{R_{3}}{q_{R_{3}}}}{K} \right)$ | *habitat restriction term* |
| $\mu$ | 0.01 (Model 2.1) 0.00 (Model 2.2) | *mutation rate (parasites* $R_{3}$ *as mutants form the host cycle* $R_{1}, R_{2})$ |
| $\alpha$ | 0.00 (Model 2.1) 0.005 (Model 2.2) | *parasite inflow per unit of time from neighboring habitat* |
| $k_{on}$ | $\left[ \begin{matrix} 0.2 & 0.19 & 0.2 \\ {10}^{-5} & {10}^{-5} & {10}^{-5} \\ {10}^{-5} & {10}^{-5} & {10}^{-5} \end{matrix} \right]$ | *parameter matrix for Model 2 (Methods or Models)* |
| $k_{off}$ | $\left[ \begin{matrix} 0.1 & 0.1 & 0.1 \\ 0.1 & 0.1 & 0.1 \\ 0.1 & 0.1 & 0.1 \end{matrix} \right]$ | *parameter matrix for Model 2 (Methods or Models)* |
| $k_{cat}$ | $\left[ \begin{matrix} 0.1 & 0.08 & 0.1 \\ 0 & 0 & 0 \\ 0 & 0 & 0 \end{matrix} \right]$ | *parameter matrix for Model 2 (Methods or Models)* |

*Model 3: (Model 3.1, Model 3.2, Model 3.0.1, Model 3.02)*

| ***Parameter*** | ***Values*** | ***Explanation*** |
| --- | --- | --- |
| $d_{R_{i}}$ | 0.005 | *decay rate (per unit of time) of ribozyme*  $R_{i}, i=1,2,3,4$ |
| $q_{R_{3}}$ | 5.0 | *factor quantizing replication efficiency of parasites* $R_{3}$*relative to host ribozymes* $R_{1},R_{2}$ |
| $q_{R_{4}}$ | 100.0 | *factor quantizing replication efficiency of hyperparasites* $R_{4}$*relative to host ribozymes* $R_{1},R_{2}$ |
| $K$ | $20.0$ | *maximum concentration of weighted sum of ribozymes* $R_{1}+R_{2}+\frac{R_{3}}{q_{R_{3}}}+\frac{R_{4}}{q_{R_{4}}}$ |
| $\Pi$ | $\left( 1-\frac{R_{1}+R_{2}+\frac{R_{3}}{q_{R_{3}}}+\frac{R_{4}}{q_{R_{4}}}}{K} \right)$ | *habitat restriction term* |
| $\mu$ | 0.01 | *mutation rate (parasites* $R_{3}$ *as mutants form the host cycle* $R_{1}, R_{2})$ |
| $\alpha$ | 0.001 | *parasite inflow per unit of time from neighboring habitat* |
| $\beta$ | 1.5 | *parameter quantizing the trigger process* $(1\&2\to3)$ |
| $k_{on}$ | $\left[ \begin{matrix} \begin{matrix} 0.2 & 0.19 \\ {10}^{-5} & {10}^{-5} \end{matrix} & \begin{matrix} 0.2 & {10}^{-5} \\ {10}^{-5} & {10}^{-5} \end{matrix} \\ \begin{matrix} {10}^{-5} & {10}^{-5} \\ {10}^{-5} & {10}^{-5} \end{matrix} & \begin{matrix} {10}^{-5} & 0.1 \\ {10}^{-5} & 0.1 \end{matrix} \end{matrix} \right]$ | *parameter matrix for Model 3.1 (Methods or Models) and Model 3.0.1 (S1 Supporting Information)* |
| $k_{on}$ | $\left[ \begin{matrix} \begin{matrix} 0.2 & 0.19 \\ {10}^{-5} & {10}^{-5} \end{matrix} & \begin{matrix} 0.2 & {10}^{-5} \\ {10}^{-5} & {10}^{-5} \end{matrix} \\ \begin{matrix} {10}^{-5} & {10}^{-5} \\ {10}^{-5} & {10}^{-5} \end{matrix} & \begin{matrix} {10}^{-5} & 1.0 \\ {10}^{-5} & 1.0 \end{matrix} \end{matrix} \right]$ | *parameter matrix for Model 3.2 (Methods or Models) and Model 3.0.2 (S1 Supporting Information)* |
| $k_{off}$ | $\left[ \begin{matrix} \begin{matrix} 0.1 & 0.1 \\ 0.1 & 0.1 \end{matrix} & \begin{matrix} 0.1 & 0.1 \\ 0.1 & 0.1 \end{matrix} \\ \begin{matrix} 0.1 & 0.1 \\ 0.1 & 0.1 \end{matrix} & \begin{matrix} 0.1 & 0.1 \\ 0.1 & 0.1 \end{matrix} \end{matrix} \right]$ | *parameter matrix for all Models 3* |
| $k_{cat}$ | $\left[ \begin{matrix} \begin{matrix} 0.1 & 0.08 \\ 0.0 & 0.0 \end{matrix} & \begin{matrix} 0.1 & 0.0 \\ 0.0 & 0.0 \end{matrix} \\ \begin{matrix} 0.0 & 0.0 \\ 0.0 & 0.0 \end{matrix} & \begin{matrix} 0.0 & 0.0 \\ 0.0 & 0.0 \end{matrix} \end{matrix} \right]$ | *parameter matrix for Model 3.1 and Model 3.2 (Methods or Models)* |
| $k_{cat}$ | $\left[ \begin{matrix} \begin{matrix} 0.1 & 0.08 \\ 0.0 & 0.0 \end{matrix} & \begin{matrix} 0.1 & 0.0 \\ 0.0 & 0.0 \end{matrix} \\ \begin{matrix} 0.0 & 0.0 \\ 0.0 & 0.0 \end{matrix} & \begin{matrix} 0.0 & 0.04 \\ 0.0 & 0.04 \end{matrix} \end{matrix} \right]$ | *parameter matrix for Model 3.0.1 and Model 3.0.2 (S1 Supporting Information)* |

*Molecular parasite:* molecular parasites constitute a (ribo)nucleotide species, which utilizes the replication machinery of hosts or of in vitro amplification reactions and typically is smaller than the host species or the amplicons that gave rise to them [2-6].

*Negative strand:* single-stranded RNA genome of opposite polarity to its original template, i.e. the single-stranded positive RNA genome.

*Parasite resistance:* when ecological conditions between the host and its parasites imply that hosts repeatedly encounter the same parasite species, a parasite-specific host response (molecular immunity) arises that increases resistance to their parasite, i.e. that reduces parasite fitness [9].

*Promiscuous*: while enzymes are traditionally believed to be specific for their substrates and the reactions they catalyze, they are called promiscuous when they diverge from this statement [10,11]. Subdistinctions are sometimes made, for instance condition promiscuity, catalytic and substrate promiscuity [10].

*Self-similarity*: sequence and particularly structural protein- and RNA-similarity increases the physical interaction propensity among self-similar molecules [12,13].

*Second-order ribozyme autocatalysis:* a ribozyme polymerase (positive strand) acts on a second template strand (negative strand) to produce a third strand (copies of the positive strand) [14].
