# Supplementary figures and images for: Biomathematical enzyme kinetics model of prebiotic autocatalytic RNA networks: degenerating parasite-specific hyperparasite catalysts confer parasite resistance and herald the birth of molecular immunity
Source: PLoS Comput Biol. 2025 Jan 3;21(1):e1012162. doi: 10.1371/journal.pcbi.1012162 (PMC11745417; doi:10.1371/journal.pcbi.1012162)

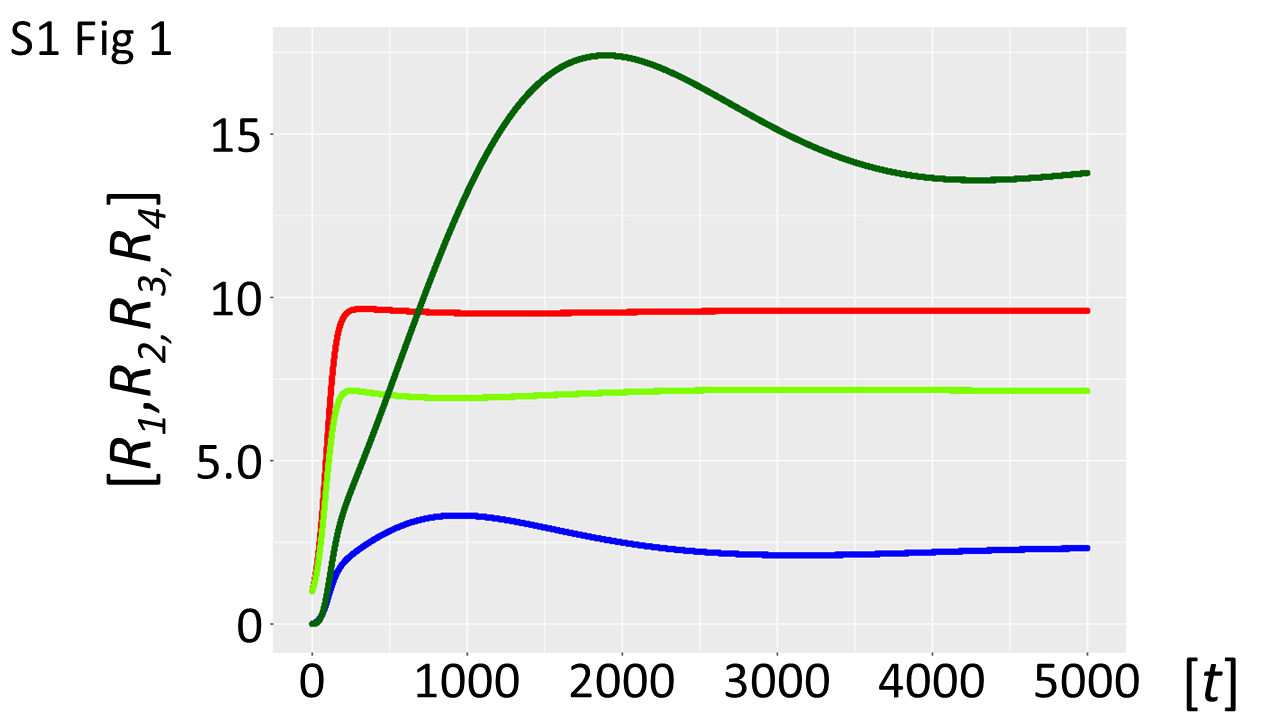

Supplement: S1 Fig — Ribozyme concentrations of defined populations R1, R2, R3 and R4 [units on unspecified scale] are plotted against time t [units on unspecified scale]. The catalytic activity is reduced (kcat 0.04 sec-1). The ribozyme polymerase R1 (red), the negative strand ribozyme R2 (light green), the parasite P (blue), and the hyperparasite F (dark green). (TIF) [file pcbi.1012162.s002.TIF]

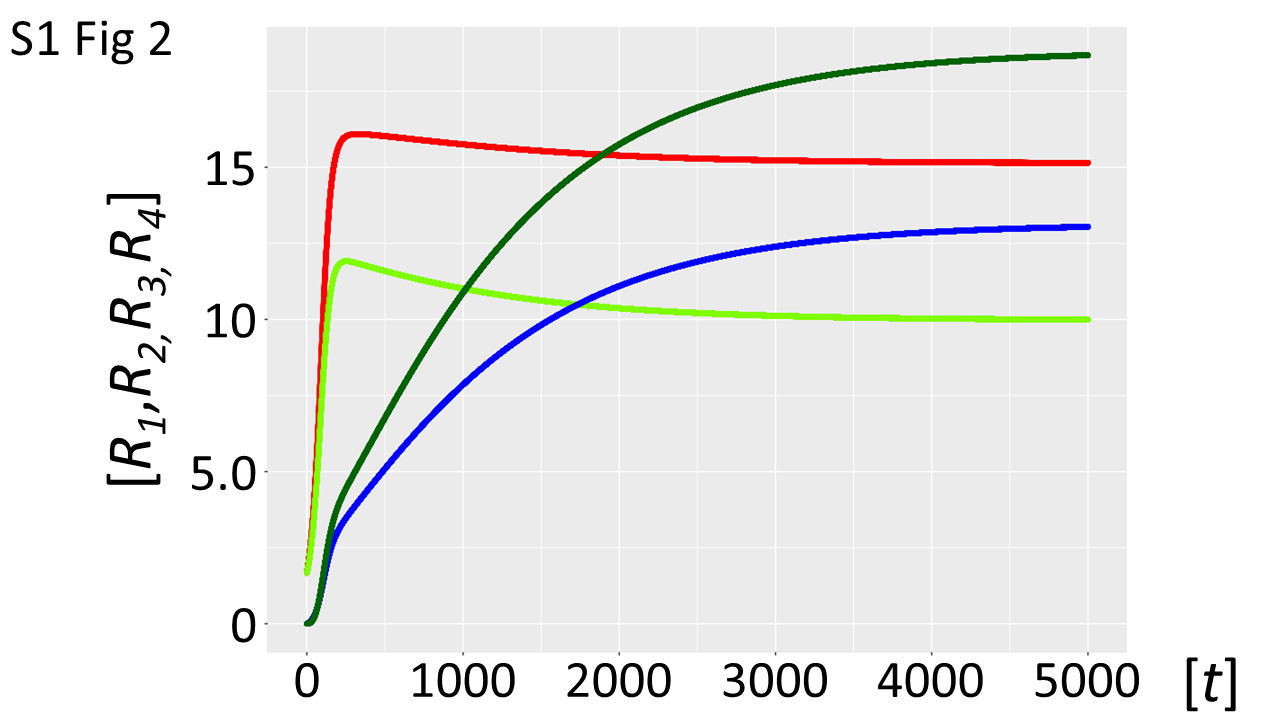

Supplement: S2 Fig — Ribozyme concentrations of defined populations R1, R2, R3 and R4 [units on unspecified scale] are plotted against time t [units on unspecified scale]. The catalytic activity of the main model is null (kcat 0.0 sec-1). The ribozyme polymerase R1 (red), the negative strand ribozyme R2 (light green), the parasite P (blue), and the hyperparasite F (dark green). (TIF) [file pcbi.1012162.s003.TIF]

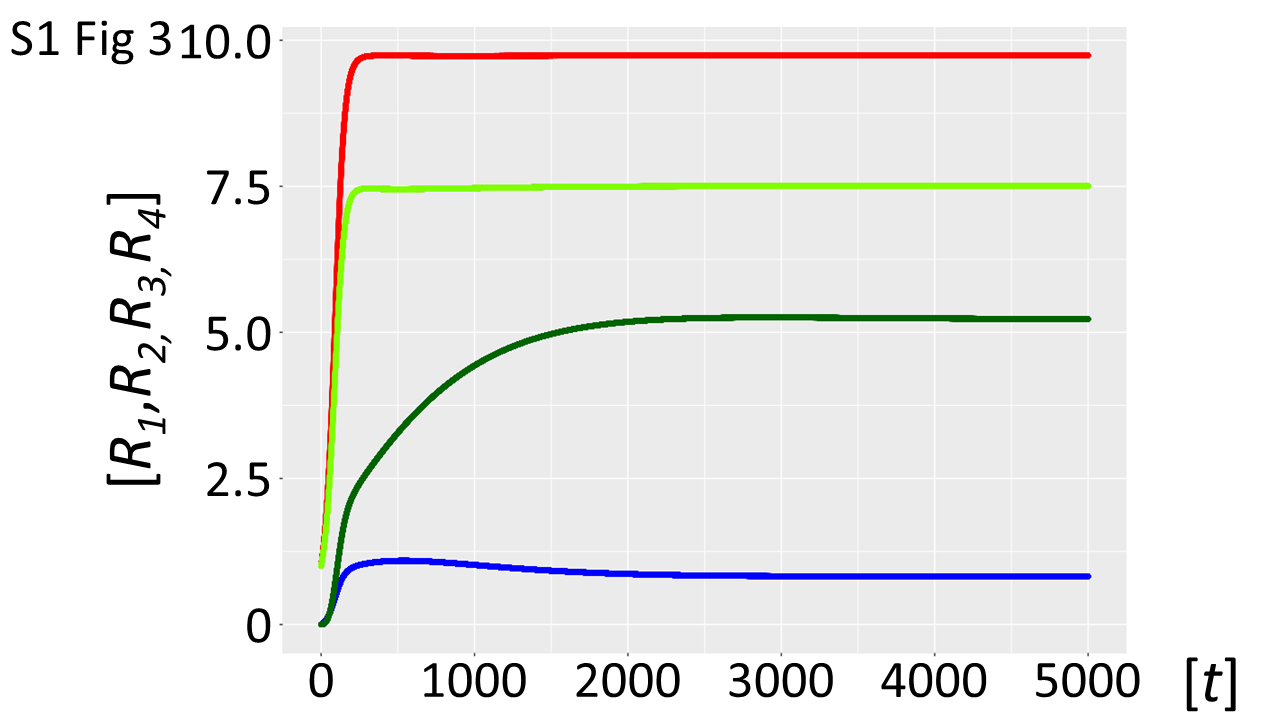

Supplement: S3 Fig — Ribozyme concentrations of defined populations R1, R2, R3 and R4 [units on unspecified scale] are plotted against time t [units on unspecified scale]. The catalytic activity is reduced (kcat 0.04 sec-1), and KM values are 0.14 μM. The ribozyme polymerase R1 (red), the negative strand ribozyme R2 (light green), the parasite P (blue), and the hyperparasite F (dark green). (TIF) [file pcbi.1012162.s004.TIF]

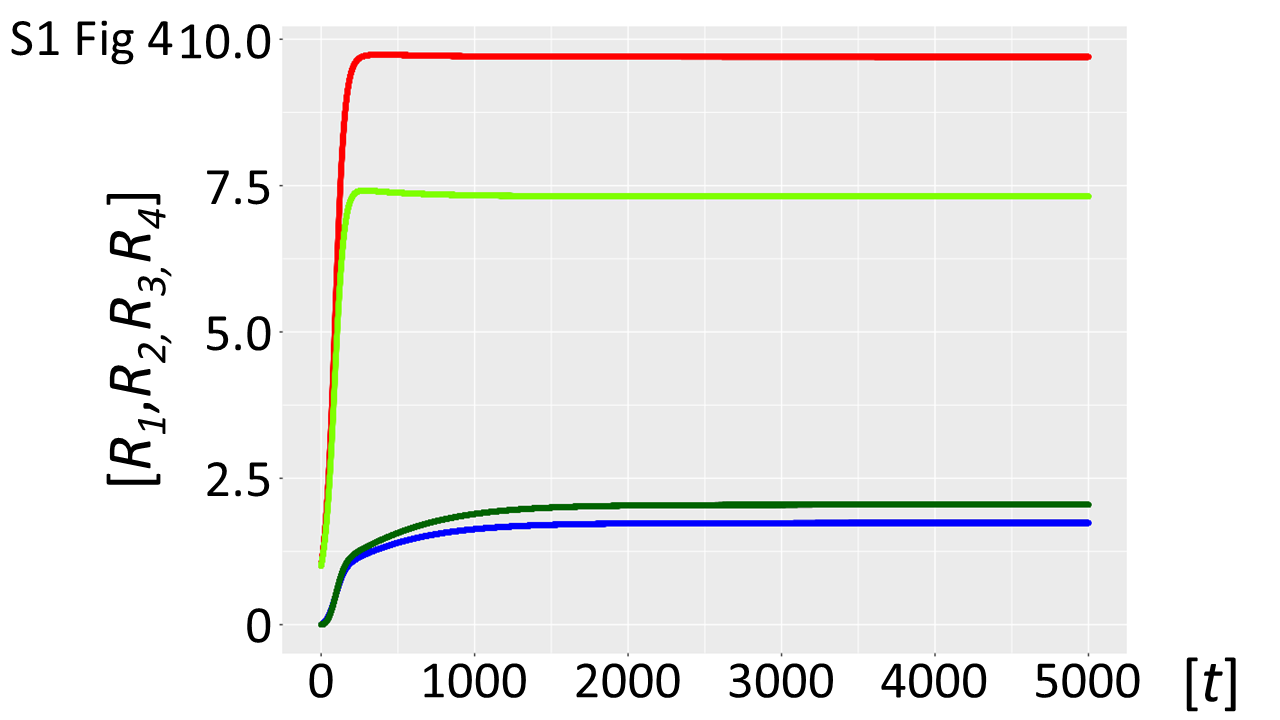

Supplement: S4 Fig — Ribozyme concentrations of defined populations R1, R2, R3 and R4 [units on unspecified scale] are plotted against time t [units on unspecified scale]. The catalytic activity of the main model is null (kcat 0.0 sec-1), and KM values are 0.14 μM. The ribozyme polymerase R1 (red), the negative strand ribozyme R2 (light green), the parasite P (blue), and the hyperparasite F (dark green). (TIF) [file pcbi.1012162.s005.TIF]
