# Supplementary material for: Biomathematical enzyme kinetics model of prebiotic autocatalytic RNA networks: degenerating parasite-specific hyperparasite catalysts confer parasite resistance and herald the birth of molecular immunity
Source: PLoS Comput Biol. 2025 Jan 3;21(1):e1012162. doi: 10.1371/journal.pcbi.1012162 (PMC11745417; doi:10.1371/journal.pcbi.1012162)
Supplement: Supplementary file 3 [file pcbi.1012162.s007.odt]

S1 Supporting Information

Authors:

Magnus Pirovino1, Christian Iseli2, Joseph A. Curran3, Bernard Conrad4*

1OPIRO Consulting Ltd., Triesen, Principality of Liechtenstein, 

2Bioinformatics Competence Center, EPFL and Unil, Lausanne, Switzerland

3Department of Microbiology and Molecular Medicine, and Institute of Genetics and Genomics of Geneva (iGE3), Geneva University, Genève, Switzerland

4Genesupport, Avenue de Sévelin 18, Lausanne, Switzerland

*[bernard.conrad@genesupport.ch](mailto:bernard.conrad@genesupport.ch)

Related work

Here, we model an alternative temporal pathway leading up to parasite resistance according to which the hyperparasite preserves a small but significant catalytic activity. This model differs in several aspects with respect to the canonical proposition presented in the main text. Firstly, primary parasite resistance here is the interlinked consequence of the higher affinity binding of F to itself and P that at the same time reduces the catalytic activity according to the accuracy-rate trade-off [1]. Selectivity (accuracy, specificity) implies the ability to discriminate between two templates, provided both are present, and rate denotes speed [1]. Ground-state discrimination means that specificity is achieved mainly through substrate binding, which imposes strong accuracy-rate trade-offs [1]. Improvements in selectivity mediated by tighter cognate substrate binding invariably lead to lower catalytic efficiency (parallel decreases in the constants for the cognate substrates KMcog and kcatcog [1]). This is precisely what the model stipulates, F has parasite- and hyperparasite-selectivity, which comes at the cost of a reduced catalytic activity that is expected, since it is a considerably smaller molecular species than the host molecules [6]. Secondly, while this solution offers superior parasite taming at the lower binding affinity level compared to the main model (compare P in blue in S1 Fig to S2 Fig), by the very nature of the approach this comes at the expense of higher hyperparasite prevalence in the habitat; latter persists at low but significant levels even after binding affinities are one order of magnitude higher (compare F in dark green in S3 Fig to S4 Fig).

We add in Model 3 further terms for the catalytic activity of : 

  

Equation  which governs the evolution of the hyperparasite   becomes now: 

Here we choosewith the same values of all other parameters (which can be freely chosen) as in Model 3.1 (Model 3.0.1 in S1 Fig).

Now, if the binding of the complexes ,  is further strengthened, by e.g. raising, and so also lowering the Michelis constants , the situation for the host cycle can even be further stabilized, with a lower population of parasites  and less molecules bound to the  and  complexes (Model 3.0.2 in S3 Fig). For comparison the main models without catalytic activity (Models 3.1 and 3.2 are shown in parallel in S2 and S4 Figs).

References

- Tawfik DS. Accuracy-rate tradeoffs: how do enzymes meet demands of selectivity and catalytic efficiency? Curr Opin Chem Biol. 2014;21: 73-80. PMID: 24954689

- Bromham L, Cowman PF, Lanfear R. Parasitic plants have increased rates of molecular evolution across all three genomes. BMC Evol Biol. 2013 Jun 19;13:126. PMID: 23782527

- Furubayashi T, Ueda K, Bansho Y, Motooka D, Nakamura S, Mizuuchi R, Ichihashi N. Emergence and diversification of a host-parasite RNA ecosystem through Darwinian evolution. Elife. 2020;9: e56038. PMID:32690137 

- Mills DR, Peterson RL, Spiegelman S. An extracellular Darwinian experiment with a self-duplicating nucleic acid molecule. Proc Natl Acad Sci U S A. 1967;58: 217-24. PMID:5231602

- Marshall KA, Ellington AD. Molecular parasites that evolve longer genomes. J Mol Evol. 1999;49: 656-63. PMID:10552046 

- Szilágyi A, Könnyű B, Czárán T. Dynamics and stability in prebiotic information integration: an RNA World model from first principles. Sci Rep. 2020;10: 51. PMID:31919467

- Bar-Even A, Milo R, Noor E, Tawfik DS. The Moderately Efficient Enzyme: Futile Encounters and Enzyme Floppiness. Biochemistry. 2015;54: 4969-77. PMID: 26219075

- Briggs GE, Haldane JB. A Note on the Kinetics of Enzyme Action. Biochem J. 1925;19: 338-9. PMID:16743508

- Paraskevopoulou S, Gattis S, Ben-Ami F. Parasite resistance and parasite tolerance: insights into transgenerational immune priming in an invertebrate host. Biol Lett. 2022;18: 20220018. PMID: 35382587

- Hult K, Berglund P. Enzyme promiscuity: mechanism and applications. Trends Biotechnol. 2007;25: 231-8. PMID: 17379338

- Copley SD. Shining a light on enzyme promiscuity. Curr Opin Struct Biol. 2017;47: 167-175. PMID: 29169066

- Lukatsky DB, Shakhnovich BE, Mintseris J, Shakhnovich EI. Structural similarity enhances interaction propensity of proteins. J Mol Biol. 2007;365: 1596-606. PMID: 17141268

- Gleitsman KR, Sengupta RN, Herschlag D. Slow molecular recognition by RNA. RNA. 2017;23: 1745-1753. PMID:28971853

- Shah V, de Bouter J, Pauli Q, Tupper AS, Higgs PG. Survival of RNA Replicators is much Easier in Protocells than in Surface-Based, Spatial Systems. Life. 2019;9: 65. PMID: 31394866
