# Supplementary material for: Biomathematical enzyme kinetics model of prebiotic autocatalytic RNA networks: degenerating parasite-specific hyperparasite catalysts confer parasite resistance and herald the birth of molecular immunity
Source: PLoS Comput Biol. 2025 Jan 3;21(1):e1012162. doi: 10.1371/journal.pcbi.1012162 (PMC11745417; doi:10.1371/journal.pcbi.1012162)
Supplement: Attachment — Submitted filename: review.pdf [file pcbi.1012162.s008.pdf]

General comment

In their manuscript, “Biomathematical enzyme kinetics model of prebiotic autocatalytic RNA networks: degenerating parasite-specific hyperparasite catalysts confer parasite resistance and herald the birth of molecular immunity”, Pirovino1 et al. investigate the mechanism underlying the emergence of life from abiotic materials. Starting with a simple enzyme kinetics model, they gradually incorporated autocatalysis, parasitism and hyperparasitism. Their analytic study of these models explains the necessity for self-amplification of genetic network within a closed loop, as well as the inevitability of both parasitism and hyperparasitism to sustain the production of genetic materials, which support the concept of host-parasite-hyperparasite trios in the context of abiogenesis.

Despite the interesting topic, I find the paper is not very reader friendly, as detailed below. I am unable to recommend a positive outcome for this version, but I would be happy to review it again once it has been revised.

Major concerns

- **Visual explanation of the research topic:** A figure like Fig 2 in S1 (in the supporting material) would be better to be in the main text. Although the topic is explained verbally, a diagram and the explanations referring it will help readers in a wider disciplines. I recommend adding model parameters and/or the name of the changing process on the arrows, in addition to the variables (i.e., R1, R2).
- **Model diagrams:** the flow charts (1.0, 2.0, 3.0 and so on) and the models (1.1, 1.2 and so on) are positioned in an appropriate order, but additional (or alternative) box that provides a comprehensive view of them all? It will be even better if it incorporates the model parameters around the arrows.
- **Clear indications of parameters and values:** What are alpha (2.3, 3.3) and beta (3.4)? Also, I recommend providing a table listing the model parameters and their meaning in this context.
- **In Method, 3. Michaelis-Menten kinetics in the RNA-world:** I didn’t find the necessity of most part of this section. Is the analytic equilibrium (Line 333) and Newton iteration (Sec. 3.3) used in this research? If not, I recommend dropping the materials irrelevant.
- **Model comparison:** The main result of this work is based on the comparison of the model simulations (Fig1-3b). The individual plots and utilized parameter values are involved in the the paper. However, I recommend arranging the figures and the used parameters side-by-side or up/down to ease the comparison.
- **Total enzyme and free enzyme mass:** the reason to define the mass of total enzyme E_{tot} must be because it is a constant, $\frac{dE_{tot}}{dt} = 0$. Indicate it in Section 2 of Method properly. In the same line, I understood R_i in Section 3 in Method are also constant. Is it correct? Also, R_i in Section 4 are the mass of free enzyme only or total enzyme?

Minor concerns

- What does nil mean? (Line 173 and so on)
- Line 288: κ_{on} and κ_{off} are exchanged.
- Line 328: drop the last ’=’
- Line 333: why only one solution of the quadratic equation?
- Line 365: $R\_R = [R_1\_R_2, ...] \to R\_R = [R_1\_R_1, ...]$
- Line 369: - $\to$ − (minus symbol is short)
- consistency of format: 0.1 or .1? (Line 522 and so on)
