# Supplementary material for: Biomathematical enzyme kinetics model of prebiotic autocatalytic RNA networks: degenerating parasite-specific hyperparasite catalysts confer parasite resistance and herald the birth of molecular immunity
Source: PLoS Comput Biol. 2025 Jan 3;21(1):e1012162. doi: 10.1371/journal.pcbi.1012162 (PMC11745417; doi:10.1371/journal.pcbi.1012162)
Supplement: Attachment — Submitted filename: PLOS Comp Biol Reviewers comments 1.docx [file pcbi.1012162.s009.docx]

**Comments to the Authors:**

**Reviewer #1:** Overall, this article is well-written, containing interesting and novel science. Qualitatively, it explores how molecular parasites seem to be an inevitable issue for autocatalytic polymers at the origins of life, but also how molecular hyperparasites seem to solve this issue, saving the original polymers from dying out due to the initial parasites. Quantitatively, the authors express these points through simulated enzyme kinetic equations of Michaelis-Menten type, employing the perpetual steady-state approximation for the concentrations of intermediate complexes. This article exemplifies the goals of this journal, combining qualitative biological insight with mathematical rigor; in particular the math is explained with great clarity.

Therefore, I would recommend it for publication, but I believe the article would benefit from some minor revisions. Specifically, a few brief updates - which I will note in detail below - would enhance the accessibility of this article to the broader community of scientists across the spectrum from biological to physical and mathematical. This article is already of high quality, and some of the following points might be addressed in included references, but the inclusion of one or two sentences here and there would make the article more self-contained:

1. The authors use biological terminology with specific meanings in this context. It would be helpful to briefly define these terms in the text, especially if the terms are central to the article, and if they could have other scientific or everyday meanings (e.g. molecular parasite, promiscuous, negative strand). This point, in particular, will assist scientists from a more physical or mathematical background understand the important qualitative results of the article.

We now provide a Text Box with formal definitions of important terms used (*Definition of terms and notations*).

2. The authors mention that they use relevant experimental values for numerical constants when available. If possible, it would be helpful to mention how the qualitative results of the article would change - if at all - when the numerical constants are varied.

The qualitative results of the model would not change if the numerical constants and parameters were varied slightly. Indeed, the robustness of settings to variation was selected to exhibit exactly that property. For example, all the parameters in Model 3 of Fig 5A can be slightly varied, yet lead qualitatively to the same result. On the other hand, Fig 5B is one example where we lowered the *K_M_* from 1.0 to 0.1 μM to show that this would lead to more efficient stabilization of the host-parasite-hyperparasite cycle and parasite taming. Nonetheless, qualitatively the results in Fig 5B are comparable to the ones in Fig 5A. We will explore in future work whether some parameters show tradeoffs towards others, for instance the affinity of different RNA molecules versus their catalytic efficiency (integrated in *Discussion*, new Lines 312-319).

3. The authors mention in the Discussion that molecular parasitism is a “chain process”, which seems to imply the existence of hyper-hyper-parasites and so on. Does the process continue to arbitrary nth-degree parasites? If so, does that affect the qualitative results of this article? If not, why does the process stop? Because the typical size of parasites gets smaller down the chain? It would be helpful to briefly clarify this question, as it seems to be a central concept to the article.

Molecular parasitism is a chain process in principle [62]. Indeed, the habitat of prototypical tripartite populations of host-parasites-hyperparasites exemplified by bacteria, phages and phage satellites contains even smaller molecular parasites that one could – at least in part – qualify as potential hyper-hyperparasites, namely a novel type of phage satellite, which is an autonomously replicating plasmid [63]. It does not integrate into the host’s genome, does not carry phage genes and is smaller than any known phage satellite so far [63]. Yet, empirical evidence suggests that tripartite populations are the prevailing habitat type [62,64]. Moreover, mobile genetic elements are highly pleiotropic, exerting multiple, sometimes contrasting effects that defy their classification as pure or even dominant parasites [63-65], (integrated in *Discussion*, new Lines 320-328).

4. The authors ordinary differential equations, encoding a deterministic time evolution of concentrations of molecular species. It could be useful for the authors to briefly address if stochastic effects would potentially alter the conclusions of their study.

The Michaelis-Menten equation is a deterministic rate equation, assuming large numbers of enzyme and substrate molecules [61]. However, with low molecule numbers, time-course measurements display large stochastic fluctuations. Unsurprisingly, stochastic and deterministic approaches may predict different numerical values for the mean substrate, enzyme and complex concentrations in time, as well as different steady-state concentrations for a given set of rate constants. These differences are typically small for the Michaelis-Menten reaction, but significant with substrate inflow [61]. Therefore, stochastic models would not significantly modify our conclusions, except for situations where external substrate provision becomes dominant (integrated in *Discussion*, new Lines 304-311).

**Reviewer #2:** General comment. In their manuscript, “Biomathematical enzyme kinetics model of prebiotic autocatalytic RNA networks: degenerating parasite-specific hyperparasite catalysts confer parasite resistance and herald the birth of molecular immunity”, Pirovino et al. investigate the mechanism underlying the emergence of life from abiotic materials. Starting with a simple enzyme kinetics model, they gradually incorporated autocatalysis, parasitism and hyperparasitism. Their analytic study of these models explains the necessity for self-amplification of genetic network within a closed loop, as well as the inevitability of both parasitism and hyperparasitism to sustain the production of genetic materials, which support the concept of host-parasite-hyperparasite trios in the context of abiogenesis.

Despite the interesting topic, I find the paper is not very reader friendly, as detailed below. I am unable to recommend a positive outcome for this version, but I would be happy to review it again once it has been revised.

**Major concerns**

• Visual explanation of the research topic: A figure like Fig 2 in S1 (in the supporting material) would be better to be in the main text. Although the topic is explained verbally, a diagram and the explanations referring it will help readers in a wider disciplines. I recommend adding model parameters and/or the name of the changing process on the arrows, in addition to the variables (i.e., R1, R2).

We moved Fig 2 S1 to the main text body, expanded it (new Fig 1A-D), and added the process names. In addition, we also provide a new Text Box (*Definition of terms and notations*), a new Fig 2 and Table 1 in the S1 Supporting Information explaining the core features and parameters of the main models, as well as of the submodels.

• Model diagrams: the flow charts (1.0, 2.0, 3.0 and so on) and the models (1.1, 1.2 and so on) are positioned in an appropriate order, but additional (or alternative) box that provides a comprehensive view of them all? It will be even better if it incorporates the model parameters around the arrows.

As suggested, we produced a new Fig 2 in the main text that provides a synopsis and flow chart of the models, with model parameters, in addition to other supplementary material, for instance a new Text Box (*Definition of terms and notations*) and Table 1 in the S1 Supporting Information, as discussed above.

• Clear indications of parameters and values: What are alpha (2.3, 3.3) and beta (3.4)? Also, I recommend providing a table listing the model parameters and their meaning in this context.

The term alpha is parasite inflow per unit of time. We added the definition for equation (2.3) in Line 515 (old Line 467), for equation (3.3) it was already defined in Line 562. We further added explicit values for alpha in Model 2.1, Fig. 2A (new Fig 4A) in Lines 873-874 and Model 2.2, Fig. 2B (new Fig. 4B) in Line 903. The term beta, the parameter quantizing the trigger process $\left( 1\&2\to3 \right),$ is defined in Line 562-563 (old Line 513). For Model 3 and all its different Figures: Fig 5A and Fig 1A-D (Model 3.1), Fig 5B and Fig 1A-D (Model 3.2, Model 3.0.1, Model 3.0.2), alpha (=0.001) and beta (=1.5) μ=0.01 are defined in Lines 562-563. The model parameters are now summarized in the new document *Definitions of terms and notations*.

• In Method, 3. Michaelis-Menten kinetics in the RNA-world: I didn’t find the necessity of most part of this section. Is the analytic equilibrium (Line 333) and Newton iteration (Sec. 3.3) used in this research? If not, I recommend dropping the materials irrelevant.

The analytic equilibrium in Line 333 (new Line 381) is called the tight-binding or Morrison equation. We could effectively omit section 3.1., since it is a well-known, simple case of a three-ribozyme system, where the equilibrium can be explicitly solved as the solution of a simple quadratic equation$\left( 3'' \right)$. Nonetheless, we think that mentioning this equation is a good starting point, leading to the more important general case of *n* ribozymes in sections 3.2 and 3.3. Here, the equilibriums for the intermediate complexes $R_{i}\_R_{j}$ cannot be solved in one explicit formula anymore. Therefore, we propose a standard Newton iteration algorithm, with an explicit formulation of its used Jacobian to solve these equilibriums. As emphasized in Line 444 (new Lines 492-493), the solution of the ODEs for all models (Model 1, 2, and 3) requires in every timestep of $t_{k}$=$t_{0}+k\Delta t$ a calculation of all the possible enzyme-template complexes $R_{i}\_R_{j}$ through the Newton iteration algorithm, comprising the relevant core of all calculations in the Figures shown.

• Model comparison: The main result of this work is based on the comparison of the model simulations (Fig1-3b). The individual plots and utilized parameter values are involved in the paper. However, I recommend arranging the figures and the used parameters side-by-side or up/down to ease the comparison.

The new Fig 2, Table 1 in S1 Supporting Information and the Text Box *Definitions of terms and notations* provide exactly what the Reviewer suggests: the individual plots and model parameters are compared side to side and up/down.

• Total enzyme and free enzyme mass: the reason to define the mass of total enzyme Etot must be because it is a constant, dEtot dt = 0. Indicate it in Section 2 of Method properly. In the same line, I understood Ri in Section 3 in Method are also constant. Is it correct? Also, Ri in Section 4 are the mass of free enzyme only or total enzyme?

The definition of the total enzyme concentration *E_tot* is needed in order to distinguish it from the free enzyme *E*. As a catalyst, *E_tot* is constant in the sense that is not changed by the process of catalyzation itself. Nonetheless, it can vary with time, either by natural decay or if *E_tot* itself is the product of catalyzation. This is explicitly the case in section 3, where the *Ri*, which are the total concentrations of ribozymes (mass), are subject to decay and reproduction processes.

**Minor concerns**

• What does nil mean? (Line 173 and so on)

*Nil* means here that the parasite species interacting with the hyperparasite $\left( P\_F \right)$and hyperparasites among them $\left( F\_F \right)$ are not productive, i.e. not producing any relevant polyribonucleotide (see also the related term *degenerate* in *Definition of terms and notations*). The underlying reason is firstly, that *P* and *F* have a marginal catalytic activity. Secondly, since *R1* mediated production of *F* is much more efficient than that potentially emanating from *P* and *F*, there is no selective pressure to maintain these processes, hence they degenerate.

• Line 288: κon and κoff are exchanged.

Done in Line 288 (new Line 335). Also done in Line 346 (new Line 394), and in *Definition of terms and notations*, Line 33.

• Line 328: drop the last ’=’

Done in Line 328 (new Line 375).

• Line 333: why only one solution of the quadratic equation?

The only relevant solution in the Morrison or tight-binding equation is the one where the concentrations of the free ribozymes (*R_1_-R_1__R_2_*) and (*R_1_-R_1__R_2_*) are nonnegative. The solution of *R_1__R_2_=1/2(R_1_+R_2_+K_M_)+sqrt*(…) would lead to negative concentrations of these free ribozymes, and is therefore of no physical relevance. We added a clarifying sentence in Line 332 (new Lines 379-380).

• Line 365: R_R = [R1_R2, ...] → R_R = [R1_R1, ...]

Done in Line 365 (new Line 413).

• Line 369: - → − (minus symbol is short)

This was corrected (Line 369, new Line 417).

• consistency of format: 0.1 or .1? (Line 522 and so on)

We now harmonized the number format throughout the manuscript (Lines 137-138, 483, 520-523, 562-565; S1 Supporting Information: Lines 57, 60, 74-76, 84-86.

**Reviewer #3:** The authors study prebiotic RNA amplification using the framework of Michaelis-Menten/Briggs-Haldane kinetics (i.e., they apply a quasi-steady-state assumption for the ODE describing the time evolution of the enzyme substrate complex). Based on the model the authors propose mechanisms how a host-parasite-hyperparasite trio can confer parasite resistance. The focus of this work is clearly not computational. The author use no methods which go beyond standard solvers for ODEs (timestep integration) and nonlinear algebraic equations (Newton iteration).

**(A) The main text is very difficult to follow.**

- The Methods Section is located at the very end of the manuscript. Unfortunately, it is impossible to understand the results without having read the Methods-Section before.

We provide a Text Box at the end of the *Introduction* with definition of important terms and notations used (*Definition of terms and notations*). We also provide new explanatory Figures positioned before the data-based Figures (Figs 1-2), and a Table 1 (S1 Supporting Information). Collectively, these efforts will greatly improve access to the core messages of the article.

- Relevant notations have to be rigorously introduced before being used, e.g., kcat, kon, koff, KM, appear first on p. 4 but are defined on p. 9

We provide a Text Box upfront in the main text with definition of important terms and notations used (*Definition of terms and notations*).

- The notation of catalyzed reactions has to be rigorously introduced (e.g., 1 ⇒(1_1) → 2 ⇒ (1_2) → 1 (1.0)). It is first used on p. 5 but formally introduced on p. 13

This has now been fixed, as discussed above.

- I suggest provide a Figure in the main text which summarizes all considered models.

As suggested, we moved Fig 2 from S1 Supporting Information to the main text, expanded it (new Fig 1A-D), and provided a new Fig 2 and Table 1 (S1 Supporting Information) featuring a synopsis of all models and their characteristics, including a workflow that interconnects the model temporally.

- Relevant termini have to be clearly defined, e.g., what is meant by “promiscuous substrate binding”

Now defined in *Definition of terms and notations.*

- Please define “degenerate”.

Now defined in *Definition of terms and notations.*

- Please define “parasite resistance”.

Now defined in *Definition of terms and notations.*

- Inconsistent cross references further complicate the understanding of the text: For an overview of the used models the authors refer to Supplementary Figure 2. This Figure visualizes 3 models, referred to as “Model 1”, “Model 2”, “Model 3”. However, in the results section the authors refer to “Model 2.1”, “Model 2.2”, “Model 3”, “Model 3.1”, “Model 3.2”, “main model” etc.

This inconsistency has now been corrected, the new Figs 1-2 and Table 1 (S1 Supporting Information) give an overview of the main and the sub-models used, including the parameters and their characteristics.

**(B) The descriptions of key results lack relevant details**

-“The catalyst accuracy-rate trade-off analysis [35] applied to autocatalytic RNA networks [34] predicts the emergence of active ribozymes [10,11,46] with binding promiscuity and high catalytic activity, co-existing with subgenome-sized molecules characteristic of parasites with relatively lower catalytic activity [40]. This is precisely what our model predicts.” Please explain why this is a model prediction. I have the feeling that it is rather an assumption put into the model (reflected in the parameter choices).

Under the parametric assumptions used (see new Table 1 and *Definition of terms and notations*), our model shows the initial emergence and self-sustenance of active ribozymes, followed by the overwhelming dominance of molecular parasites causing the habitat to collapse. All available models empirically rooted in biophysics and theoretical considerations convergently predict that molecular parasites will be smaller than host molecules and at least initially catalytically less active [37-40]. In conclusion, the results of our model are consistent with both empirical evidence and theoretical consideration of molecular parasite biology. We have rephrased the sentence that now reads “Our model is fully consistent with these predictions” (new Lines 255-256). In addition, the new Table 1 (S1 Supporting Information) opposes assumptions and results from the underling theoretical framework for all models and submodels.

- “In conclusion, with growing sequence divergence of host and parasite catalysts the probability of self-binding increases, and the trend towards cross reactivity diminishes, precisely as suggested by our model.” Please describe in detail how this conclusion can be deduced from the model and under which assumptions. As far as I understand the model does not explicitly account for sequence similarity and its quantitative impact on physical interactions. Therefore, I am puzzled how the authors obtain this specific mechanistic conclusion from a rather phenomenological modeling approach.

Our model achieves homeostatic stability under the assumption that the two classes of host and parasite/hyperparasite molecule species preferentially interact among themselves and do not show significant cross-reactivity. Empirical biophysical evidence shows that this is likely to happen, both for protein and RNA templates [42,45]. As discussed above, the model is consistent with predictions that are strongly supported by experimental evidence. We have now rephrased “….our model is fully consistent with” (new Line 210). In general, the limitations of conclusions drawn are discussed in the new Table 1 (S1 Supporting Information), and new Lines 282-285).

-“In sum, these simulations show that parasite resistance can be modelled with biochemical
precision, and it relies on hyperparasites acquiring parasite binding specificity, via at least two
temporally distinct pathways.” This conclusion sounds odd. The authors do not compare results to data, therefore it remains unclear to me what they mean by “biochemical precision”.

We disagree, increasing parasite resistance is indeed modelled with biochemical precision: i) the presence of hyperparasites *per se* confers a certain stability (i.e. host molecules will survive in time), when assuming *K_M_* values reported for ribozymes (Model 3.1), ii) or stipulating in accordance with experimental data [35] that hyperparasites lose some but not all catalytic activity (Model 3.0.1). However, definitive parasite taming as depicted in models 3.1 and 3.0.1, in which the host now displays higher and more stable prevalence relative to the parasitic templates, is only brought about by increasing the *k_on_* rate, which leads to *K_M_* values that are one order of magnitude lower (Fig 2 and Table 1 in S1 Supporting Information).

- A main argument of the proposed reasoning is that the hyperparasite F has a very high specifity for the parasite P and that P_F complexes from with a higher probability compared to F_R1 complexes. Please elaborate on the question why such a constellation is more probable to arise during evolution compared to a situation where the parasite is highly specific for the host and P_R1 complexes from with a higher probability compared to P_F complexes. I agree with the conclusion “As long as these
hyperparasites acquire parasite binding-specificity, an increasingly solid and molecularly specific
parasite resistance will ensue” However, I do not see why this scenario is probable to arise during molecular evolution.

As discussed in this *Rebuttal* in Lines 243-249, self-similarity among protein and RNA molecule species predicts not only an increased probability of physical interaction, but also a higher binding affinity [42,45]. In conclusion, as shown experimentally [37] host and parasitic populations diverge molecularly yet retain self-similarity with their starting templates, and therefore it is expected that self-reactivity will increase with the extent of their sequence and structural-spatial divergence.

- All simulation results are dimensionless which makes it very difficult to interpret them and to compare the different considered scenarios.

As pointed out above, the parameters are dimensionless in principle, but whenever solid experimental parametric data was available, we used them and modelled as discussed with biochemical precision (see particularly also new Fig 2 and Table 1 in S1 Supporting Information).

-“the abundance of these two molecular species increases rapidly”. What does “rapidly” mean? The simulation provides no time unit.

We disagree, as shown (former Fig 1, new Fig 3) in 250 arbitrary time units, host ribozymes attain a consistent prevalence that remains stable in time. In a direct comparison and using an identical timescale (new Figs 4A and 4B), parasites attain a plateau much slower (in 1250 and 750 arbitrary time units).

- The caption of each figure should provide information about the used parameters

This is now systematically provided.

- “Figure 1 shows a graphical representation of this model assuming realistic starting kinetics and habitat restriction parameters (described in Methods or Models).” Please elaborate on what “realistic” means or refer the reader to a specific subsection of the Methods part where this issue is discussed.

We now refer to specific subsections in *Definitions of terms and notations*.

- Fig 2A: How do the dynamics look like on a longer time scale. Does the system approach an equilibrium state?

As mentioned in the text body, the habitat under these conditions will collapse because it is overwhelmed with parasitic molecules that determine their own demise; they literally “dig their own grave”. In practice and as expected, *R_1_* and *R_2_* were already undetectable at the arbitrary timepoint-unit 2000, and *P* strongly diminished from 1250 to 2000. This trend is fully confirmed in the new Figs 4A^1^ and 4B^1^at timepoint 5000.

- “As a corollary, F will be a highly efficient template, yet a poor or non-functional ribozyme capable
of self-amplification.” Please elaborate on this statement. Does the same reasoning also apply to P?

As mentioned in the text body and based on experimental evidence [37-40], shorter parasitic templates tend to be more efficiently amplified (i.e. they are better templates), but have lower catalytic activity (inferior ribozymes), while longer templates have more elaborate spatial structures that improve catalysis (superior ribozymes; [40]), yet negatively interfere with replication (worse templates). Consequently, this applies to both *P* and *F*, which is also symbolized in the new Fig 1, in the sense that *F* will be smaller than *P* (better template and inferior catalyst).

- “Secondly, in order for hyperparasitism to be highly efficient, it is required that F has a high specificity for P; i.e. in the presence of both R1 and P it binds mainly or exclusively to the latter” Please justify this statement in more detail and provide a formal proof in terms of the proposed models.

This aspect has already been addressed in two previous sections of this *Rebuttal* (Lines 243, 272), it pertains to self-similarity. The hyperparasite *F* is more similar to *P*, from which it derived than from the host template, and accordingly *F* will interact with and have a higher binding affinity for *P* than for the host templates *R_1_* and *R_2_*.

**(C) More detailed explanations could significantly improve the readability of the text.**

- “However, this process is not subject to evolutionary pressure and is bound to degenerate ( ⇒ (_) → , ⇒ (_) → (3.0)).” Please explain this statement in more detail.

This notion pertains to parasite degeneration that is now formally defined in *Definition of terms and notations*. *Nil* means that the interactions between parasites and hyperparasites (*P_F*), as well as interactions among hyperparasites themselves (*F_F*) are not productive, i.e. not producing any relevant polyribonucleotide. The underlying reason is firstly, that *P* and *F* have marginal catalytic activity. Secondly, since *R1* mediated production of *F* is much more efficient than that potentially emanating from *P* and *F*, there is no selective pressure to maintain these processes, hence they degenerate.

- l. 189 what is meant by “and the level of P remains important.”

As can easily be seen in the new Fig 2 and Table 1 in S1 Supporting Information (previously Figs 3A and 3B, and in S1 Supplemental Information Figs 1A-D) parasite taming materializes in two steps. In a first phase the prevalence of the host molecules *R_1_* and *R_2_* is restored to levels similar to Model 1 at the outset. However, and this is the statement made here, *P* and *F* remain prevalent. Only in the second phase is definitive parasite taming achieved (Models 3.2 and 3.0.2), suppressing *P* and *F* to near background levels, at least in the main Model 3.2 (Fig 2 and Table 1 in S1 Supporting Information).

- Supplement: Bottom of page 10: Why is the solution R1_R2=1/2(R1+R2+KM)+sqrt(…) not considered?

The only relevant solution of the Morrison or tight-binding equation is the one where the concentrations of the free ribozymes (*R_1_-R_1__R_2_*) and (*R_1_-R_1__R_2_*) are nonnegative. The solution of *R_1__R_2_=1/2(R_1_+R_2_+K_M_)+sqrt*(…) would lead to negative concentrations of these free ribozymes, and is therefore of no physical relevance. We added an additional clarifying sentence in Line 332 (new Lines 379-380).

- I do not see how the procedure described in section 3.3 differs from what is realized by standard solvers provided by any numerical software.

The Newton iteration is a standard procedure to find the solution of the multidimensional nonlinear problem of equation$\left( 3 \right)$, a system of $n^{2}$ equations with $n^{2}$ unknown ribozyme-template complexes $R_{i}\_R_{j}$. We found it useful to explicitly write down the solving algorithm with the respective formula for the specific Jacobian matrix needed here.

- Please justify the algebraic expression of the habitat restriction constant or provide a suitable reference.

The habitat restriction term was introduced in our initial model [31] based on Lotka-Volterra equations. Previously, Koonin et al. defined it in an analogous context as carrying capacity [16].

- The parameter choices presented in the supplement have to be justified / motivated in more detail.
We have given these considerations ample space. We provide an additional Text Box (*Definition of terms and notations*) that summarizes the parameter choices for all models.

**(D) I miss a section which systematically relates the model output to data or to experimental observations.**
This is now accomplished in the new Table 1 in S1 Supporting Information that lists the main and sub-models, the assumptions, the model results, as well as the underlying theoretical framework supporting it.

**(E) Parasitism and hyperparasitism have been studied for a long time. I miss a systematic comparison to previous works and a paragraph summarizing the key novelties of this work.**

We now provide an extended comparison and discuss the key novelties of our model (new Lines 286-303).

**(F) The abstract has to be reworked and clearly distinguish between model assumptions and drawn conclusions. Colloquial terms should be replaced by rigorous scientific language.**

As suggested, the abstract has been reworked to distinguish model assumptions and conclusions, and undefined terms have been eliminated.

**Minor**
-“A complete autocatalytic replication cycle comprises the initial production of R2 by two R1 ribozyme molecules, and the subsequent reproduction of R1 by R1 using template R2” Please provide a reference supporting this mechanism

Previous work by Higgs et al. [23,28] uses an identical setup: the antisense *R_2_* that is catalytically inert is produced by two *R_1_* ribozymes and serves a template for *R_1_* amplification (see also new Lines 292-294, *Definition of terms and notations*).

-“For a number of reasons they 148 are inseparably associated with all life forms [16,17], with the most important being the accuracy-rate 149 trade-off [34,35].” Please explain this statement in more detail, e.g., in the introduction section

Although the arguments relative to the accuracy-rate trade-off have been developed on four separate occasions, we understand that an introduction is also required. This is what we have done in the new *Definition of terms and notations*.

-“In general, enzymes operate orders of magnitude (kcat/KM of ∼105 M–1s–1) below the
150 diffusion rate (108-109 M-1s-1). This implies promiscuous substrate binding, high catalytic activity, and as 151 a secondary trade off, a high mutation rate generating a large variance in the quasispecies: i.e. speed 152 being more important than enzyme accuracy [36].” Please explain this statement in more detail, e.g., in the introduction section

As mentioned above, although this notion has been addressed repeatedly, we understand that it also needs to be represented in the Introduction. This is what we have done in the new *Definition of terms and notations* at the end of the *Introduction*.

-“Indeed, this is precisely what 178 the accuracy-rate trade-off predicts, with selectivity implying the ability to discriminate between two 179 templates when both are present [35], whereas rate denotes speed [35,36].” This senetence is hard to digest.

We added the formal definition of the accuracy-rate tradeoff (*Definition of terms and notations*).

-“Ground state 180 discrimination means that specificity is achieved mainly through substrate binding, which imposes 181 strong accuracy/rate trade-offs [34,35].” Please explain this statement in more detail, e.g., in the introduction section

We added the formal definition of the *ground-state discrimination* and related terms in *Definition of terms and notations*.

- “Improvements in selectivity mediated by tighter cognate 182 substrate binding invariably leads to lower catalytic efficiency (parallel decreases in the constants for the cognate substrates KM
cog and kcat 183 cog [34,35]).” Please explain this statement in more detail, e.g., in the introduction section

We added the formal definition of the *ground-state discrimination* and related terms in *Definition of terms and notations* at the end of the *Introduction*.

- Fig. 3A “This already happens if intermediate complexes P_F and F_F have a KM in the μM range” To facilitate the interpretation please state the KM values of all catalytic processes involved in Model 3. The same applies to Fig 3B.

This has now been accomplished in the new Fig 2 and Table 1 in S1 Supporting Information.

- “Given what is currently known 197 about template selection, modulating the on-rates appears to be the favoured option [43,44].” Please shortly explain why

Given the paramount importance of the four-step formation of an active polymerase elongation complex [43], accelerating association rates appears the favorite option.

- “We subsequently confirmed earlier models 228 showing that these autocatalytic cycles are sustainable, as long as a sufficient nucleotide pool is 229 available.” Please provide references to the “earlier models”.

We added the two missing references (new Line 235).
